# Development of a Core Outcome Set for Studies on Cardiac Disease in Pregnancy (COSCarP): a study protocol

**DOI:** 10.1186/s13063-020-04233-1

**Published:** 2020-03-30

**Authors:** Rohan D’Souza, Chelsea Hall, Mathew Sermer, Samuel Siu, Candice Silversides

**Affiliations:** 1 Department of Obstetrics & Gynaecology, Division of Maternal-Fetal Medicine, Mount Sinai Hospital, University of Toronto, 3-908 – 700 University Avenue, Toronto, Ontario M5G 1Z5 Canada; 2 Division of Cardiology, Schulich School of Medicine and Dentistry, Western University, London, Canada; 3 Department of Medicine, Division of Cardiology, Obstetric Medicine Program, Mount Sinai Hospital, Toronto, Canada

**Keywords:** Core outcome set, Cardiac disease, Pregnancy, Patient-reported outcomes, Qualitative research, Delphi Survey, Outcome reporting

## Abstract

**Background:**

Clinical studies looking at interventions to optimize pregnancy and long-term outcomes for women with cardiac disease and their babies are inconsistent in their reporting of clinical outcomes, making it difficult to compare results across studies and draw meaningful conclusions. The development of a core outcome set (COS)—a standardized, minimum set of outcomes that must be collected and reported in all studies—is a practical solution to this problem.

**Methods/design:**

We will follow a five-step process in developing a COS for studies on pregnant women with cardiac disease. First, a systematic literature review will identify all reported outcomes (including patient-reported outcomes) and definitions. Second, semi-structured interviews with stakeholders involved in the care of pregnant women with cardiac disease will determine their perspective and add new outcomes that they consider important. Third, an international electronic Delphi survey will narrow outcomes obtained through the first two steps, in an attempt to arrive at a consensus. Fourth, a face-to-face consensus meeting will deliberate to finalize the COS. Finally, measurement tools and definitions for included outcomes will be determined through a series of literature reviews and Delphi surveys.

**Discussion:**

This protocol provides an overview of the steps involved in the development of a COS that must be reported in studies involving pregnant women with cardiac disease, in an attempt to harmonize outcome reporting and ensure the validity of study results that will not only inform clinical practice and future research but also encourage the development of COS in other areas of medicine.

**COMET core outcome set registration:**

http://www.comet initiative.org/studies/details/834

## Background

It is estimated that approximately 1–4% of pregnant women have some form of congenital or acquired cardiac disease [1]. Advances in pediatric cardiology and cardiac surgery have led to higher numbers of women with congenital heart disease reaching reproductive age [2], while increasing maternal age and medical comorbidities have contributed to the rise in acquired heart disease among pregnant women worldwide. According to the most recent review into maternal and child health in the United Kingdom, cardiac disease continues to be the leading cause of indirect maternal mortality both during and up to 6 weeks after pregnancy [3]. Similar reports from the United States [4] and from low/middle income countries [5] suggest that, despite the success of safe motherhood initiatives, cardiovascular disease is the leading cause of maternal mortality from non-obstetric causes in both high- and low-income countries. In high-income countries, where maternal mortality rates are lower, these pregnancies remain at increased risk for serious maternal and fetal morbidity [1].

The past few decades have seen a rise in well-designed, prospective studies to address unanswered questions concerning the management of these pregnancies [6]. However, recruiting pregnant women into clinical trials is a challenge, and the number of patients with a particular cardiac condition seen in each tertiary centre continues to remain small. As a result, we continue to be reliant on meta-analyses of results from smaller observational studies to draw meaningful clinical conclusions and guide practice. Meta-analyzing data requires that outcomes are reported and measured/defined consistently. This unfortunately is not the case. We also have almost no information on what maternal and fetal/neonatal outcomes are considered important by pregnant women with cardiac disease. Standardization of outcome measurement and reporting and incorporation of patient input on the value of reported outcomes are the two major challenges in appropriate translation of research knowledge to good clinical practice. The development of a core outcome set (COS) can address these challenges and, in doing so, reduce bias in outcome reporting, enable meta-analysis of published data to inform decision-making, and provide an empiric basis for inclusion of stated outcomes based on input from relevant stakeholders [7].

A COS comprises a list of outcomes that is systematically derived, incorporates preferences of patients and other stakeholders involved in their care, and is required to be collected, measured, and recorded as a baseline in all clinical studies for a given disease or condition [8]. We present a protocol for the development of a COS for studies on cardiac disease in pregnancy (COSCarP), based on recently outlined principles by the Core Outcome Measurement in Effectiveness Trials (COMET) initiative [7], and in compliance with the Core Outcome Set—Standardized Protocol Items (COS-STAP) statement [9]. The scope of COSCarP is to develop a single COS that can be used in all observational and experimental studies that involve the management of pregnant women with any form of congenital or acquired cardiac disease.

## Methods/design

The COS is being developed under the auspices of the Outcome Reporting in Obstetric Studies (OROS) group, an international collaboration, led by the University of Toronto, Canada, dedicated to ensuring the standardization and comprehensiveness of outcome reporting in obstetric studies [10]. COSCarP was registered on the COMET website after checking the COMET database and confirming that there were no overlapping projects [11].

### Step I: Systematic review

A systematic literature review will be undertaken with the primary aim of identifying all reported outcomes in studies involving pregnant women with cardiac disease and generating a preliminary list of outcomes that are reported by researchers. The primary research question is “what maternal and fetal/neonatal outcomes have been reported in studies involving pregnant women with congenital or acquired heart disease” and the secondary question is “how have these reported outcomes been defined and/or measured?” The protocol for this systematic review, based on the Preferred Reporting Items for Systematic Reviews and Meta-Analyses (PRISMA) guidelines, is available on the international prospective register of systematic reviews, PROSPERO (CRD42016038218) [12]. COS-specific considerations are outlined below.

#### Study selection

Three bibliographic databases—Medline, Embase, and Web of Science—will be searched from inception. Given the limited availability of studies and lack of randomized controlled trials (RCTs) in this patient population, we will include all prospective and retrospective experimental and observational studies.

#### Data extraction

Extracted information will include details on study characteristics such as publication year, journal, country and study setting, number of participants, study type, number of included pregnancies and type of cardiac lesions, information on interventions, as well as individual and composite outcomes and their definitions, components, and measurement instruments.

#### Quality assessment

As the purpose of this review is to identify reported outcomes and not to determine the effectiveness of management strategies, no assessment of the study’s methodological quality will be performed. The quality of outcome reporting has, in the past, been assessed using parameters identified by The Management of Otitis Media with Effusion in Children with Cleft Palate (MOMENT) group [13]. By these criteria, each study receives a numerical score ranging from 1 to 6, with points assigned to clearly stating and defining the primary and secondary outcomes, describing the rationale for selecting outcomes, and a subjective assessment of the quality of outcome measurement; a score above 4 out of 6 is said to indicate good reporting quality. However, these criteria are developed for randomized trials, which make a distinction between primary and secondary outcomes, while most studies done in pregnant women with cardiac disease are not randomized trials, and therefore do not make this distinction. Second, these criteria do not take into account the reporting of both maternal and fetal/neonatal outcomes, which are vital to studies in pregnant women [14]. Third, the description of reporting quality is not considered a pivotal component of systemic reviews for COS development, that are aimed at determining all published outcomes, regardless of study quality. Finally, the criteria published by the MOMENT study group have not been validated. For these reason, as with the more recent systttematic reviews for the development of COS, assessment of the quality of outcome reporting will not be performed.

#### Analysis and presentation of results

The proportion of studies reporting each outcome and the components and definitions of each composite outcome will be documented. Although most outcomes relevant to the broad area of cardiac disease in pregnancy are similar, some outcomes are specific to certain types of cardiac disease. For example, the major concern in pregnancies with mechanical heart valves is related to maternal thromboembolic complications and fetal congenital malformations as a result of the primary condition and major bleeding from the use of blood thinners to manage the primary condition [15]. In women with cardiac transplants on the other hand, the risks are primarily related to graft rejection and premature birth [16]. Similarly, aortic dissection is a life-threatening complication but is mostly relevant to patients with aortopathies, while surgical risks and outcomes related to recovery from surgery are only relevant to those undergoing cardiac interventions during pregnancy. Based on agreement between the COSCarP investigators, with years of experience in managing patients with cardiac disease, we divided the broad clinical area of cardiac disease into six distinct subgroups: valvular heart disease, cardiomyopathies, complex congenital heart disease, aortopathies, arrhythmias, and cardiac interventions during pregnancy. Subgroup analyses will be performed based on the subgroups of cardiac lesions to identify outcomes that are specific to some cardiac conditions and not to others. This step will play an important role in presenting the final COS, as described further in step IV.

### Step II: Stakeholder interviews

It is being increasingly recognized that conducting interviews with stakeholders identifies unique outcomes that are seldom, if at all, reported in trials [17]. Previous studies by the OROS group showed that although clinical outcomes and, to some extent, adverse events are adequately reported in the medical literature studies, the areas least represented include those related to functioning/life-impact and resource use [18]. Interviews with stakeholders provide the perfect opportunity to elicit outcomes related to these domains, to add to the list of (mostly clinical) outcomes obtained through systematic review.

We will invite two groups of individuals to participate in semi-structured interviews to identify clinical health outcomes that they consider important: (1) current and former patients, patient representatives, and patient advocates, and (2) stakeholders directly or indirectly involved in the care of pregnant women with cardiac disease. Recognizing that healthcare professionals in different roles and from different clinical settings would be able to add unique perspectives, the latter subgroup would include cardiologists, maternal-fetal medicine physicians, obstetricians, obstetric medicine physicians, obstetric and cardiac anesthesiologists, midwives, family physicians, obstetric and cardiac nurses, neonatologists and ultrasonographers, researchers, hospital administrators, guideline developers, and policy-makers. Patients, patient representatives, and patient advocates will be recruited in person through the cardiac disease and pregnancy clinic at Mount Sinai Hospital, Canada’s largest tertiary referral center for high-risk pregnancies, situated in Toronto, one of the most multicultural cities in the world. This hospital has one of the largest cardiac disease in pregnancy programs and cares for women of diverse cultural, ethnic, and socio-economic backgrounds with a wide range of congenital and acquired heart disease. Although outcomes considered in studies on most subtypes of cardiac disease are similar, as described earlier, some outcomes may be specific to certain subtypes of cardiac disease, and we will aim to recruit patients with a diverse range of conditions with varying severity. Conducting face-to-face or phone interviews with patients from this center would add a sufficiently representative sample of patient-reported outcomes to the list of outcomes gathered from the systematic review, ahead of the international Delphi survey, which would ensure representation of patients’ perspectives from around the world. Other stakeholders will be recruited through email via contact lists assembled by the study investigators. In doing so, we will try to ensure representation from clinicians and clinician-researchers from internationally renowned centers that routinely manage pregnancies with cardiac disease.

Each interview will begin with participants filling in a brief demographic questionnaire that identifies their stakeholder group and, when relevant, their area of specialization, years of experience caring for pregnant women with cardiac disease, heart condition, and whether they had any complications during their pregnancies. After a brief introduction, and providing a description of the project and explanation on what constitutes health outcomes, participants will be asked to list pregnancy-related health outcomes they deem most significant and provide a rationale for their selecting those outcomes. Series of interviews will be conducted until no new outcomes are identified in two successive interviews, referred to as “saturation”. Stakeholders that are unable or unwilling to attend an in-person interview will be given the option of a telephone interview. Interviews will be recorded and analyzed by qualitative researchers and the information obtained from the discussions will be presented. The specifics with regard to data collection and analysis will follow the principles outlined in our protocol for the development of a COS for pregnant women with increased body weight [19]. With regard to the COS, step I and step II will provide a comprehensive list of outcomes for the next step in COS development.

### Step III: The Delphi process

The Delphi process, which is designed to achieve convergence of opinion in an iterative and sequential manner, will follow published principles specific to COS development [20]. The COSCarP steering committee, which comprises the study investigators, will be supported in this step by members of the OROS project [10], which includes researchers, clinicians, graduate students, and patient representatives. The OROS group will work closely with the COSCarP investigators to ensure continued input from all groups of stakeholders during the entire process. We aim to conduct two rounds of surveys to arrive at consensus, the operational details of which are outlined below.

#### Developing the survey

The long list of outcomes generated by steps I and II will be reviewed by experts comprising healthcare providers, patients, and qualitative researchers with a view to creating a shorter list of outcomes by removing redundancies, stratifying outcomes into those common to all cardiac diseases and those specific to certain sub-categories, and grouping outcomes into domains based on a recently published taxonomy [21]. Lay-language summaries will simultaneously be created that will appear alongside the complex medical outcomes. The survey will initially be piloted through face-to-face interviews with a representative sample of patients and other stakeholders in order to receive input on structuring of survey items, survey length, and the adequacy of lay-language summaries.

#### Identifying Delphi panel members

The approved survey will be distributed to members of all stakeholder groups using DelphiManager™ software, to ensure privacy, feasibility, cost effectiveness, and reliability [8], while facilitating global representation. The recruitment and identification of potential participants will occur through a multitude of channels in an endeavor to ensure inclusive representation: 1) patients and advocates through cardiac disease in pregnancy clinics, patient-advocacy groups, social media platforms, and mother–baby blogs; 2) researchers through author lists of papers included in the initial systematic review; 3) clinicians through member lists of relevant national societies and participant lists published by international conferences and steering committee members’ contacts; and 4) other stakeholders through author lists of published guidelines and known administrators of healthcare units. This would ensure global representation. Investigators will review demographic questionnaires during the course of the Delphi survey and target recruitment towards the geographical areas and cardiac disease subtypes that are under-represented, to ensure that representation is as global as possible.

There are currently no recommendations with regard to a sample size for a Delphi survey. However, COS developed for obstetric conditions have recruited between 15 and 32 patients and 68–194 professionals into the first round, of which 0–25 patients and 37–149 professionals have completed the final round of the Delphi survey. Based on these numbers, and in order to try and ensure as equal representation of patients as healthcare professionals as possible, we will aim to recruit at least 50 patients and 50 professionals from the other stakeholder groups. We will try and ensure that patients with the six subtypes of cardiac disease are represented in the Delphi Survey, by targeting them through pregnancy-and-heart disease blogs and other social media platforms.

#### Obtaining consent and demographic details

The link provided to Delphi panel members will specify the survey’s purpose, echo the importance of participation in both rounds and provide instructions that participants would use as a reference throughout the process. Panel members would be required to electronically sign a consent form and fill out a brief questionnaire to provide information on which of the four stakeholder groups they primarily identify with, their specific role (e.g., current or former patient, research nurse, cardiologist, etc.), age, country of residence, sex, and experience with COS development. With the exception of the stakeholder identification, which is essential to ensure adequate representation and to presenting results by group, for subsequent rounds, all other questions on the questionnaire will be voluntary.

#### The Delphi process

Upon completion of the demographic questionnaire, participants will be required to score each outcome on a nine-point Likert scale based on degree of importance as recommended by the Grading of Recommendations Assessment, Development and Evaluation (GRADE) working group [22]. Scores of 1 to 3 will be considered as “not essential”; 4 to 6, “important but not critical”; and 7 to 9, “critically important for inclusion”. Participants will have the option of selecting “unable to score” if they feel they lack exposure or experience to adequately score a given outcome. Outcomes that are ranked “not essential” (score 1–3) by > 70% of all stakeholder groups will be removed. For the remaining outcomes, mean scores will be calculated first for all participants and then for stakeholder groups. These results will be presented graphically to panel members at the time of the second round of the survey along with their assigned score, in a few weeks after the first. Participants will have the option of adding new outcomes that were not included in the initial survey. New outcomes emerging during round 1 will be reviewed by the study investigators to determine whether they are truly unique, i.e., not represented in whole or in part by an existing outcome. If unique, these outcomes will be included in round 2. Recognizing that these will only have been scored once (in round 2), all these outcomes and their scores by stakeholder group will be carried forward to the consensus meeting, where they will be discussed.

In the second round, panel members will again be required to score each extant outcome but will have the option of either adhering to their previous score or changing their score based on the new information provided. Outcomes deemed “critically important” by ≤ 15% of each stakeholder group and “not essential” by ≥ 70% will be removed, and those deemed “critically important” by ≥ 70% and “not essential” by ≤ 15% will be included in the final COS [7]. The rest of the outcomes will be discussed at the consensus meeting. At the second round of the survey participants will be informed of a face-to-face consensus group meeting to finalize the COS and asked if they would like to participate. It has been suggested that ranking of outcomes in the final step of the survey might help with reducing the number of outcomes, but given the large number of maternal and fetal outcomes under the “clinical/physiological” core area, ranking could result in these outcomes, albeit rare, replacing the “less severe” patient-reported outcomes, although more common and important to patients, in the final COS. This phenomenon has been observed with other COS developed for obstetric conditions. Our plan for ensuring true representation of important outcomes in the final COS is described under step IV.

One of the biggest challenges with Delphi surveys is attrition rates between rounds and ranges between 21% and 48% in published COS [23]. Strategies that have been employed to maximize response rates and reduce attrition bias include offering a 6-week window to complete each round of the survey, outlining expectations at the onset, and weekly e-mail reminders. We will also consider extension of the survey’s deadline and sending personalized reminders in an attempt to reduce attrition rates.

### Step IV: Consensus meeting

Two to five Delphi panel members from each stakeholder group that expressed interest in participating will be randomly selected to attend the face-to-face consensus group meeting wherein debate amongst stakeholder groups will help determine the final set of core outcomes. This consensus meeting will occur over a half or full day (depending on the number of outcomes to be discussed), and will be moderated as an open discussion by an expert in qualitative research, using the nominal group technique [24]. Herein, participants are asked to first independently respond to questions posed by a moderator, then discuss answers within small groups, and finally prioritize ideas or suggestions from all group members, thereby reflecting the group’s overall preferences. Given the need for participants to be present in person for this event, participants, who will be reimbursed for their time and travel, will need to be randomly drawn from the Greater Toronto Area. Since international representation has already been sought through the Delphi survey, and since we will be using a cautious approach to eliminating outcomes, we do not see local representation of stakeholder groups at this stage as compromising the study’s integrity.

The specialty of obstetrics involves two distinct individuals—mother and baby—and decisions made during pregnancy often have long-term impacts on the lives of both individuals. It is not surprising, therefore, that the numbers of outcomes that must be reported in obstetric trials are larger than in other areas of medicine. The OROS project, under whose initiative COSCarP is being developed, endeavors to achieve a balance between standardization and comprehensiveness of outcome reporting. While the development of a minimum set of outcomes addresses the former, the latter is also important to ensure representation of outcomes related to both mother and baby and inclusion of outcomes from all core areas, as identified in the medical taxonomy of outcomes for clinical trials [21], which include mortality/survival, clinical/physiological, life-impact/functioning, resource-use, and adverse events. There will, therefore, be no limit on the final number of outcomes. All outcomes deemed “critically important for inclusion” (score 7–9 on the Delphi survey) by both patients and other stakeholders, as well as only by patients (but not by other stakeholders), will be retained and included in the final COS. This will include surrogate outcomes and multi-attribute outcomes.

The purpose of the consensus meeting will not be to overrule any consensus achieved through the Delphi process, or attempt to condense the number of core outcomes to a pre-specified number, as stated above, but to agree on inclusion of three main groups of outcomes: (1) those deemed “critical but not important” by other stakeholders, but not by patients, (2) those deemed “important but not critical” by one or both groups of participants, and (3) new outcomes that emerged at round 1 and only received participant input in one round (round 2). Items not scored by all participants (missing data) will also be discussed at the meeting to arrive at agreement on whether the failure to score these items by participants within certain groups could result in inadvertent exclusion.

Since most outcomes in studies related to cardiac disease in pregnancy are common to all subtypes of heart disease, and many studies include all types of cardiac disease as a single group, we will develop a single COS for all cardiac diseases in pregnancy. This being said, some outcomes—many of which are life-threatening— remain very specific to certain subtypes of cardiac disease, as will be determined by the subgroup analysis in step I. At the consensus meeting, study investigators will draw on the data from the subgroup analysis in step I to mark these outcomes with an asterisk and provide explicit instructions on which subtype of cardiac disease they are relevant to.

The core outcomes identified through the above process will, therefore, be presented in tabular form, separating maternal from fetal/neonatal outcomes, each stratified by the five main core outcome areas, with an asterisk against those outcomes that are only relevant to certain study types. A supplementary table highlighting all outcomes, their Delphi scores, and the stages at which they were excluded will also be presented for greater transparency.

### Step V: Determination of outcome definitions and measurements

Upon selection of a final list of core outcomes, we will employ the COnsensus-based Standards for the selection of health Measurement INstruments (COSMIN) to assess each measurement tool/ definition for these included outcomes based on four criteria: validity, responsiveness, reliability, and interpretability [25]. Depending on the final list of selected outcomes, it might be necessary to conduct systematic reviews and/or Delphi surveys involving relevant stakeholder groups to determine the most appropriate definition or measurement instrument for each identified core outcome [26]. A protocol specific to this stage will be developed following the completion of step IV and is beyond the scope of this protocol.

### Knowledge translation

COSCarP findings will be presented at international obstetrics and cardiology meetings, published in an open-access journal and archived in the COMET and CoRe Outcomes in Women’s Newborn Health (CrOWN) databases.

## Discussion

Cardiac disease in pregnancy is the leading cause of maternal mortality in both high- and low-income countries. Understandably, the outcomes reported in these studies are often life-threatening to both mother and baby. However, in addition to these life-threatening outcomes, which are fortunately rare, are numerous patient-reported outcomes which affect the quality-of-life of a greater proportion of these women. These tend not to be reported in clinical studies, although they are important to patients, presumably because they are not considered as serious as some of the other clinical outcomes. An important focus of the OROS group in developing COSCarP is to ensure that patient-reported outcomes, which are underrepresented in published studies, stand a chance of being incorporated in the final COS while ensuring that outcomes related to both mother and baby are reported in future studies. This manuscript describes our protocol for the development of a COSCarP which will provide researchers studying the effectiveness of interventions for cardiac disease and pregnancy with an empiric basis for inclusion of the stated outcomes, which in turn is based on input from patients with cardiac disease and stakeholders involved in their care. We anticipate that COSCarP will reduce bias in outcome reporting and assist authors of systemic reviews to appropriately meta-analyze data to guide clinical decision-making. It is our hope that principles we have outlined in this protocol will promote the development of COS in other areas of clinical research where standardization of outcome reporting remains a concern.

## Data Availability

Not applicable.

## Abbreviations

COS: Core outcome set
COSCarP: Core Outcome Set for Studies on Cardiac Disease in Pregnancy
COMET: Core Outcome Measurement in Effectiveness Trials
CROWN: CoRe Outcomes in Women’s and Newborn Health
COSMIN: Consensus-based Standards for the selection of health Measurement Instruments
COS-STAP: Core Outcome Set—Standardized Protocol Items
